# Modulation of Chemotherapy Sensitivity of Breast Cancer Cells through Transforming Growth Factor-beta Pathway-mediated Alterations in DNA Damage Response

**DOI:** 10.7150/ijms.111217

**Published:** 2025-03-31

**Authors:** Abdullah S. Alhamed, Mohammad S. El-Wetidy, Mervat M. Abdelwahed, Sabry M. Attia, Abdulrahman M. Alabkka, Saleh A. Alaraj, Khalid Alhazzani, Ahmed Z. Alanazi, Faris Almutairi, Ibrahem A. Alotibi, Mohammed Alqinyah

**Affiliations:** 1 Department of Pharmacology and Toxicology, College of Pharmacy, King Saud University, Riyadh 11451, Saudi Arabia.; 2 College of Medicine Research Center, College of Medicine, King Saud University, Riyadh 11461, Saudi Arabia.

**Keywords:** TGF-β signaling pathway, nucleotide excision repair, cisplatin, breast cancer

## Abstract

Chemotherapeutic drugs, like cisplatin, function by damaging genomic DNA, thus inducing cell apoptosis. Cancer cells can enhance their DNA repair capacity, leading to chemotherapeutic resistance. Nucleotide excision repair (NER) involves repairing DNA adducts and crosslinks caused by chemotherapeutic agents. Transforming growth factor-beta (TGF-β) pathway contributes to carcinogenesis, DNA repair alteration, and chemoresistance. However, the connection between TGF-β pathway, NER function alteration, and resistance to cisplatin therapy remains elusive. Therefore, the objective of current study was to fill this gap by assessing the impact of TGF-β inhibition and activation on cisplatin-induced antiproliferation, apoptosis, and DNA damage using the MTT assay, flow cytometry analysis, and COMET assay, respectively. Four NER genes, XPA, XPB, XPC, and XPF, were measured using Real-time Polymerase Chain Reaction (qPCR). MDA-MB-231 cell line was utilized as a model of breast cancer. Blockade of the TGF-β pathway strengthened cisplatin cytotoxicity, whereas induction of the TGF-β pathway suppressed cisplatin cytotoxicity. In cisplatin-treated breast cancer cells, DNA damage significantly increased upon the TGF-β pathway inhibition. Conversely, cisplatin-induced DNA damage decreased significantly upon TGF-β pathway stimulation. Finally, cisplatin caused an overexpression of the four NER genes which was curtailed and augmented by TGF-β inhibition and stimulation, respectively. Overall, this study presented evidence of the impact exerted by TGF-β pathway on NER and cisplatin sensitivity of breast cancer cells.

## Introduction

Breast cancer persists as a complicated disease which is associated with high rates of morbidity and mortality globally 1. Based on cellular and molecular features, breast cancer has been categorized into various clinical groups that significantly influence the treatment outcomes of breast cancer 2-4. Treatment of breast cancer includes chemotherapy, which is a fundamental and effective approach, especially for triple-negative breast cancer. However, chemoresistance and subsequent metastasis are frequently observed in patients with breast cancer 5. The advancement in cancer treatment has improved the clinical outcomes, but treatment failure has continued to challenge cancer therapy. Therefore, development of new logical approaches to cancer research and therapy is urgently needed to overcome treatment failures.

Most chemotherapeutic agents primarily exert their action via inducing DNA damage, which can stall the transcription and DNA replication. Cisplatin is a commonly used chemotherapeutic agent to treat various malignancies, such as lung, colorectal, breast, testicular, and ovarian 6. Cisplatin causes DNA lesions, intra- and interstrand crosslinks, via creating covalent bonds between cisplatin and DNA bases which are repaired by the NER 7. DNA damage caused by other chemotherapeutics drugs, including cyclophosphamide and doxorubicin can also be repaired by NER, in cooperation with other pathways of DNA repair. The normal cell response to such insults is to remove the DNA lesions, which can ultimately lead to cell survival 8. In the tumor microenvironment, the increase in DNA repair pathways is considered a mechanism of drug resistance, leading to less effective cancer treatment using genotoxic agents 9.

NER is a DNA repair pathway that consists of two main DNA repair pathways. One of these pathways is global genome NER that follows a sequential order of activation, involving recognition of the damaged site via XPC-RAD23B complex, unwinding the double helix structure around the damaged DNA oligomer by specific helicase (XPB and XPD), removal of the DNA lesions by endonucleases XPF-ERCC1 and XPG, and eventually synthesis of new DNA oligomer at the site of the removed DNA lesions 7. NER is significantly involved in maintaining genomic instability and protecting against cancer, as shown in the cancer-prone syndrome, xeroderma pigmentosum. Dysregulation of NER has been associated with chemotherapeutic resistance, although the mechanism of this dysregulation is still unknown 8,10.

TGF-β is a multifunctional polypeptide that prominently controls cell proliferation, immune response, and wound healing. When activated, TGF-β receptors stimulate downstream signaling of Smad-dependent and Smad-independent pathways. Under normal physiological conditions, TGF-β inhibits cellular growth and differentiation and acts as a tumor suppressor 11. Several diseases are linked with abnormality of TGF-β pathway, including various cancer types 12. Dysregulated TGF-β signaling can also promote cancer development processes, resulting in enhanced tumor growth and therapy resistance 13-15.

The contribution of the TGF-β pathway in inducing chemotherapeutic resistance through modulation of NER has not been investigated yet. However, the TGF-β pathway altered the response to DNA damage upon exposure to ionizing radiation and chemical carcinogens 14,16-19. The focus of the present study is to explore the link between TGF-β pathway and breast cancer cells' response to cisplatin and whether the DNA repair, specifically the NER pathway, is involved in this process. Therefore, this study has the potential to uncover novel therapeutic targets in the treatment of breast cancer.

## Materials and Method

### Cell culture and reagent

In this study, MDA-MB-231 (ATCC, Virginia, USA) was utilized which was originally established from pleural effusion of human metastatic breast cancer. Cells were grown in Dulbecco's Modified Eagle's Medium (DMEM) with added mixture of penicillin-streptomycin antibiotic (1%) and fetal bovine serum (10%) purchased from Thermo Fisher Scientific.

Cisplatin, LY2109761 (TGF-β receptor blocker), and SRI-011381 hydrochloride (TGF-β signaling agonist) were procured from MedChemExpress (New Jersey, USA). LY2109761 and SRI-011381 have been widely utilized as agents to modify TGF-β signaling pathway in the investigation of various disease models 20-22. In our preliminary pilot study, we determined that the optimal concentrations for a robust therapeutic response were 88 µM cisplatin, 2.5 µM LY2109761, and 10 µM SRI-011381. RT and SYBR Green qPCR Master Mixes were procured from MedChemExpress (New Jersey, USA). All primers were acquired from Integrated DNA Technologies (Leven, Belgium).

### Cell proliferation assay

MTT was employed to assess the proliferation of MDA-MB-231 cells. Cells (2.5x10^4^ per well) were seeded on a 96-well plate for 24 hours. Next, cells were treated with cisplatin, LY2109761, and SRI-011381 for 24 hours. Upon treatment end, 10 µL of MTT solution (5 mg/ml) was pipetted into the wells and left at 37 °C for three hours followed by discarding the medium containing MTT and adding 100 µL dimethyl sulfoxide. The samples' absorbance at a 570 nm wavelength was finally measured using BioTek microplate reader (Elx-800, USA).

### Apoptosis assay

Cells were seeded overnight in an appropriate cell culture condition followed by treatment with cisplatin, LY2109761, and SRI-011381 for 24 hours. Afterwards, cells were harvested and washed twice with 1X PBS. Following the manufacturer's protocol, the Biolegend FITC Annexin V for apoptosis detection containing propidium iodide (PI) was used to evaluate apoptosis. A BD FACSCalibur (BD Biosciences) was used to examine the stained samples.

### COMET assay

The COMET assay was employed to analyze DNA damage in each experimental group. In brief, 100 µL of agarose (0.5%) with low melting temperature was mixed with one million cells which was then spread on cold glass slides precoated with 1.5% melting agarose and left to solidify on ice for 10 minutes. An additional 90 µL low-melting agarose was added on the slides for 10 minutes on ice and then transferred to slide jars containing cooled lysing solution and left in the refrigerator overnight. After incubation, slides were immersed in an ice-cold electrophoresis solution with pH > 13 for 30 minutes before conducting electrophoresis for 30 minutes at 300 mA and 4 °C. Following electrophoresis, 0.4 M Tris neutralization buffer (pH 7.5) was added three times to wash the slides. Each slide was then stained by adding 50 μL of ethidium bromide solution (20 μg/mL) for 5 minutes before rinsing with water. Then, slides were analyzed within 4 hours using a fluorescent microscope (Nikon, Japan) equipped with appropriate filters. The experiments were run in triplicate with duplicate slides for each experiment for a total of six slides per condition. For each slide, a total of one hundred cells were assessed using COMET assay IV software from Perceptive Instruments (Suffolk, UK). The percent of tail intensity was used as a parameter to assess DNA damage.

### Real-time polymerase chain reaction

Total RNA was extracted using TRIzol reagent (Invitrogen) according to manufacturer protocol. cDNA was synthesized using RT Master Mix and the qPCR was performed using SYBR Green. XPA, XPB, XPC, XPF, and GAPDH forward and reverse primers were utilized (Table 1). The calculation was done using the method of 2^-ΔΔCT^ with GAPDH as an endogenous housekeeping control. The fold difference between groups was displayed relative to control.

### Statistical analysis

The GraphPad Prism 9 was used to generate the figures and to compute the statistics. A one-way ANOVA and *post hoc* Tukey's tests were carried out to ascertain the statistical significance between multiple groups. A p<0.05 was deemed statistically significant. Results were displayed graphically as a mean ± SEM.

## Results

### TGF-β inhibition using LY2109761 potentiated cisplatin-induced apoptotic and antiproliferative actions

**Figure 1:**
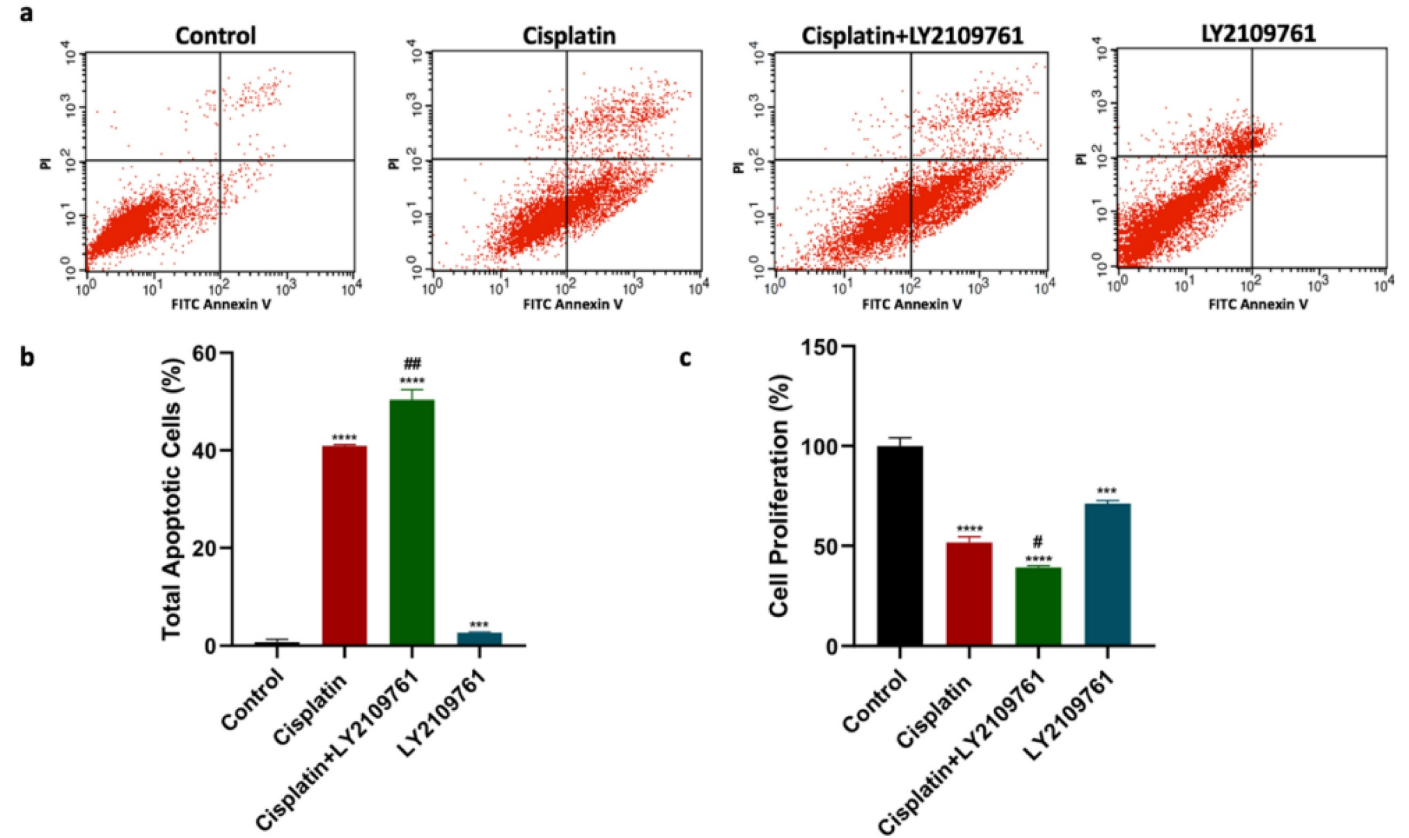
** Influence of LY2109761 on cisplatin-induced apoptotic and antiproliferative effects. (a)** Cell apoptosis histograms of stained samples following 24-hour of cisplatin ± LY2109761 treatment.** (b)** Total cell apoptosis (%) after 24 hours of treatment. **(c)** Percentage of cell proliferation after 24 hours of treatment. # and* represented statistical significances compared to cisplatin and control, respectively. ^#^ p < 0.05, and ^##^ p < 0.01, *** p < 0.001, and **** p < 0.0001.

The apoptotic assessment was carried out to determine the impact of TGF-β blockade on cisplatin cytotoxic effects. Compared to untreated breast cancer cells, cisplatin treatment significantly augmented total cell apoptosis (Fig. 1a and 1b). Cotreatment of cisplatin and LY2109761 significantly boosted the total apoptosis compared to cisplatin alone. The total cell apoptosis of breast cancer cells was significantly increased by treatment with LY2109761 alone compared to untreated breast cancer cells. Additionally, MTT assay was performed to investigate the influence of TGF-β blockade on the proliferation of breast cancer cells. The cell proliferation was suppressed by cisplatin compared to untreated cells (Fig. 1c). Combination of LY2109761 and cisplatin significantly improved the cisplatin-induced decrease of cell proliferation. When compared to untreated cells, LY2109761 alone exerted a significant antiproliferative effect.

### TGF-β inhibition using LY2109761 increased the amount of DNA damage induced by cisplatin

**Figure 2:**
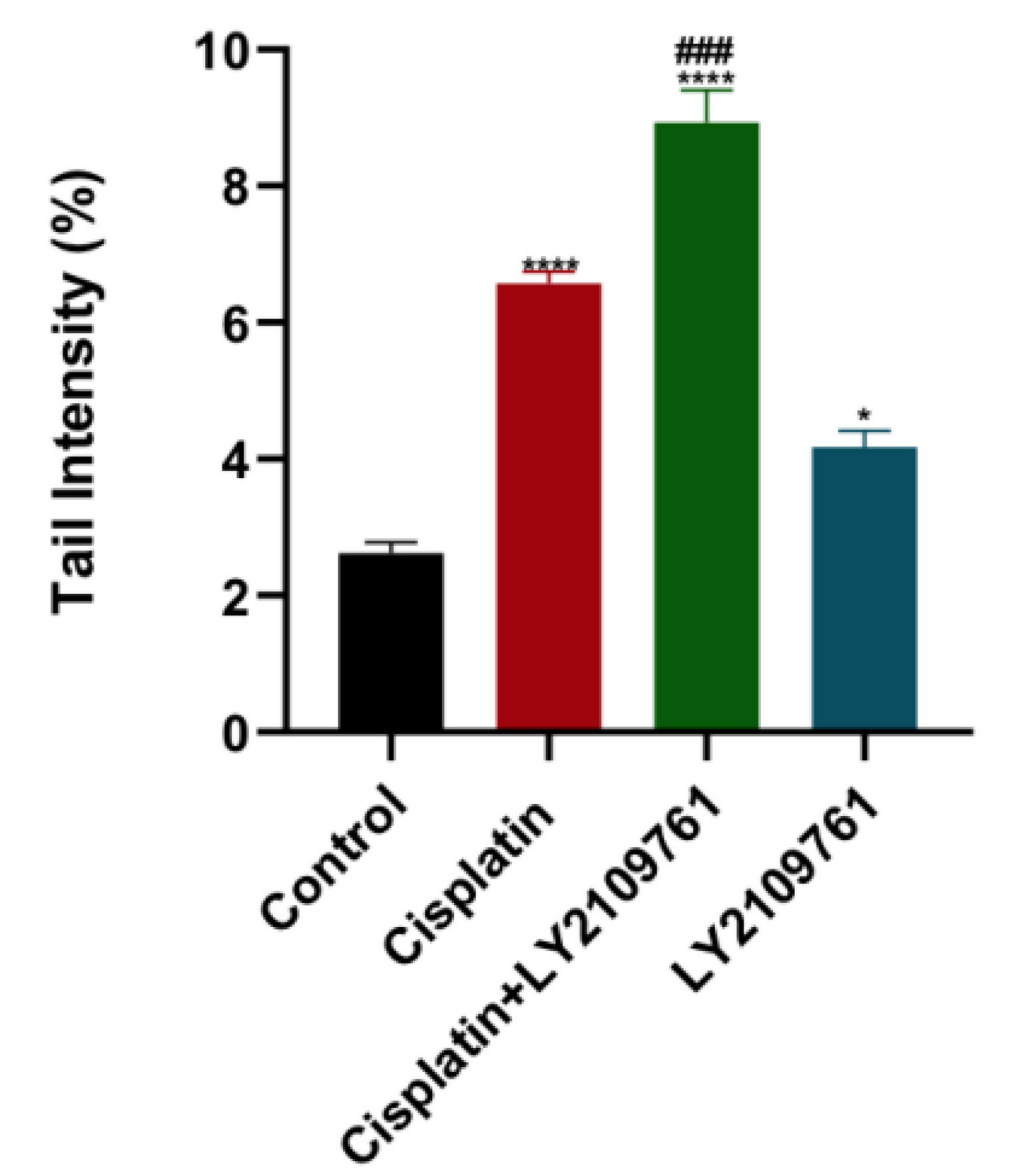
** Influence of LY2109761 on cisplatin-induced DNA damage.** Amount of DNA damage in breast cancer cells represented by the tail intensity (%) after treatment with cisplatin ± LY2109761 for 24 hours. # and* represented statistical significances compared to cisplatin and control, respectively. ^###^ p< 0.001, * p< 0.05, and **** p< 0.0001.

To evaluate the impact of TGF-β blockade on DNA damage caused by cisplatin, COMET assay was utilized to measure DNA damage which is represented by the tail intensity. DNA damage in cisplatin-treated cells was significantly higher than untreated cells (Fig. 2). Adding LY2109761 to cisplatin resulted in more cellular DNA damage than cisplatin alone. LY2109761 alone significantly elevated cellular DNA damage.

### TGF-β inhibition using LY2109761 suppressed the NER gene expression caused by cisplatin

**Figure 3:**
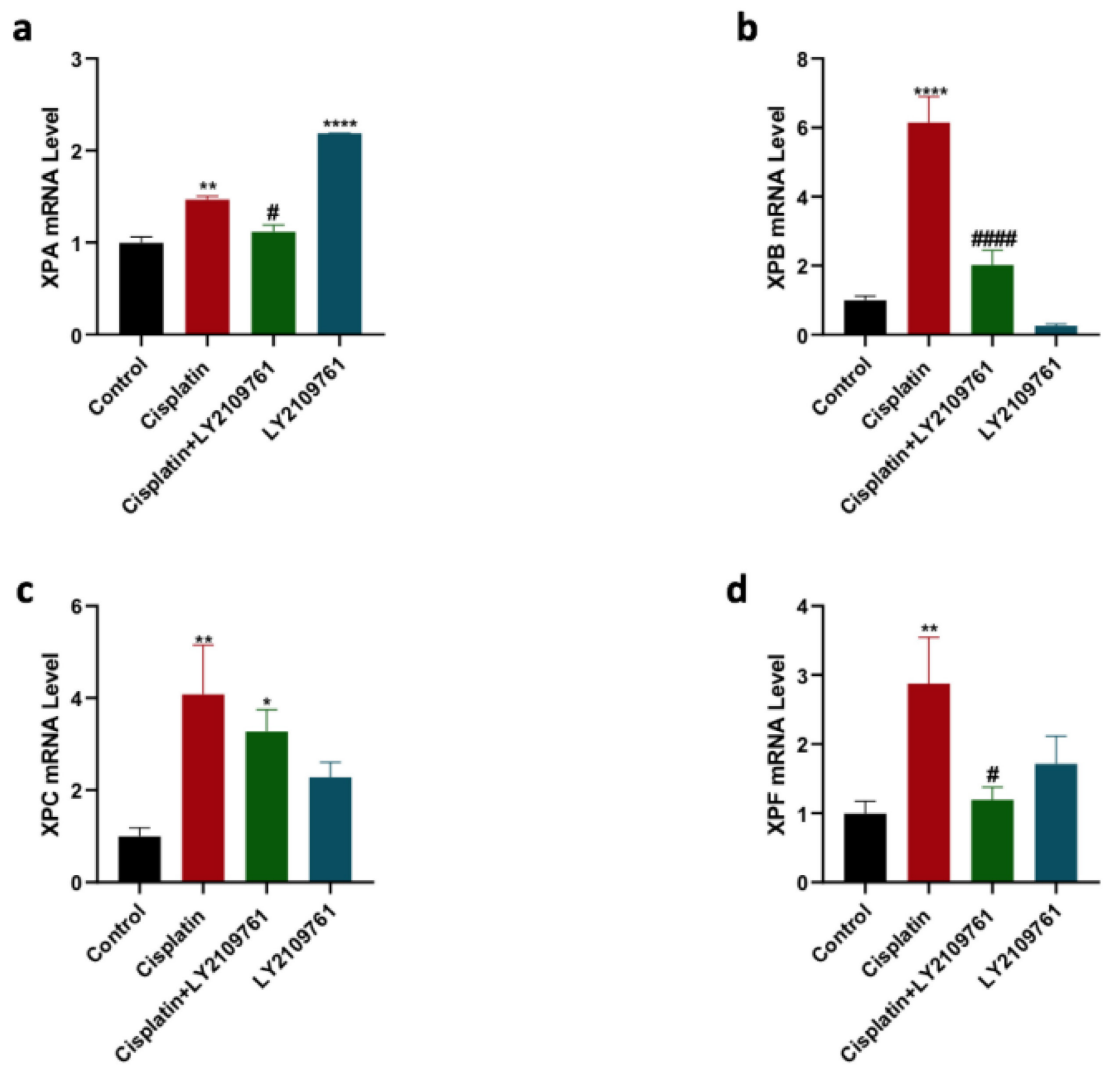
** Influence of LY2109761 on the expression of NER genes induced by cisplatin therapy.** Expression of XPA, XPB, XPC, and XPF genes (Figure 3a-d) in cells treated with cisplatin ± LY2109761 for 24 hours. # and* represented statistical significances compared to cisplatin and control, respectively. ^#^p< 0.05, and ^####^ p < 0.0001, *p< 0.05, ** p< 0.01, and **** p< 0.0001.

mRNA level of four NER genes, including XPA, XPB, XPC, and XPF, was measured to analyze the influence of TGF-β blockade on cisplatin-induced increase in the expression of these NER genes. Cisplatin therapy caused a significant overexpression of XPA, XPB, XPC, and XPF genes (Fig. 3 a-d). The upregulation in the expression of XPA, XPB, XPC, and XPF genes in cisplatin-treated group was reduced by combining cisplatin with LY2109761, which was statistically significant for XPA, XPB, and XPF genes. LY2109761 alone did not affect the gene expression of XPB, XPC, and XPF in comparison with the control. Surprisingly, the XPA gene was significantly elevated by LY2109761 monotherapy.

### TGF-β activation using SRI-011381 attenuated apoptotic and antiproliferative actions of cisplatin

**Figure 4:**
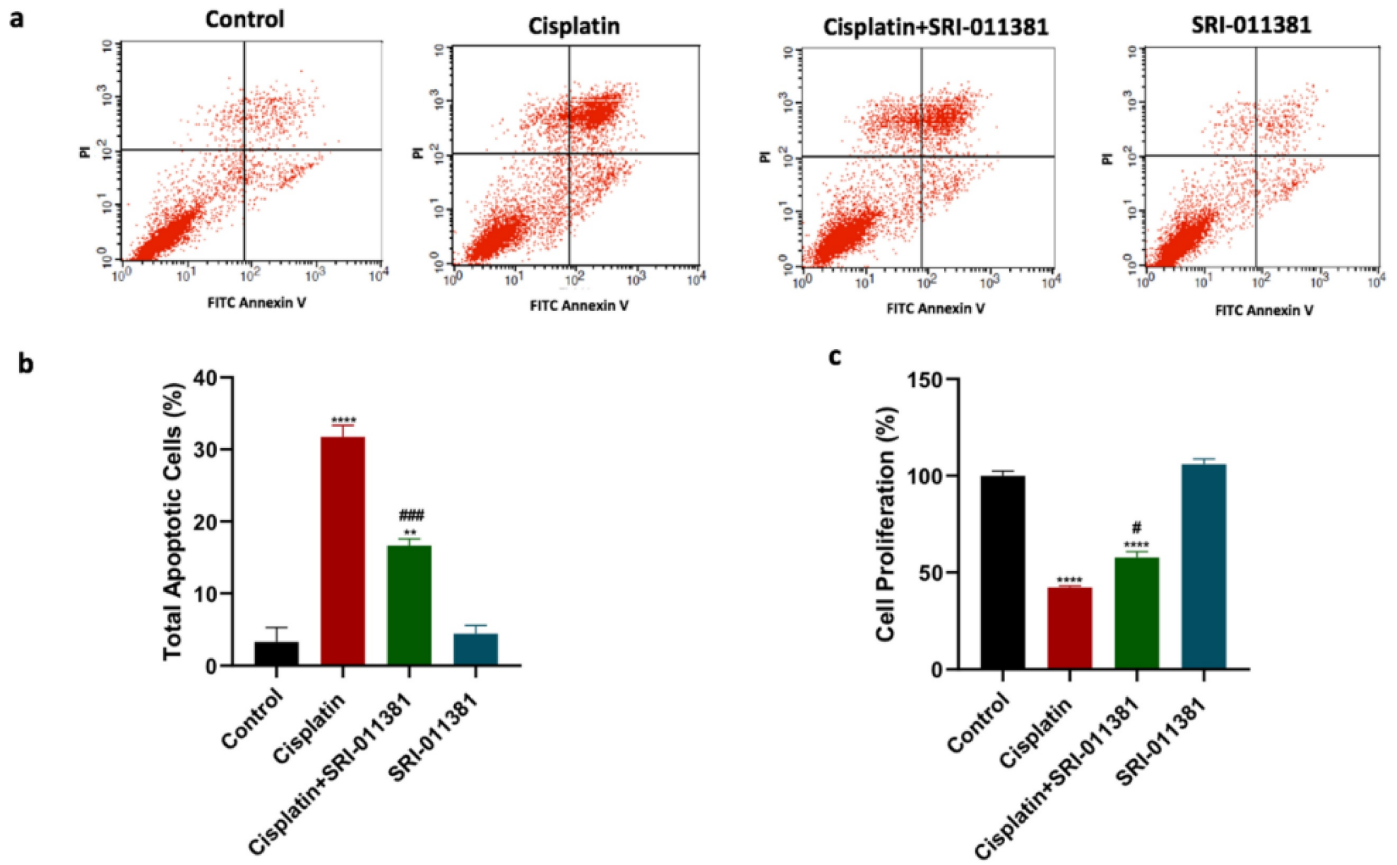
** Influence of SRI-011381 on cisplatin-induced apoptotic and antiproliferative actions. (a)** Cell apoptosis histograms of stained samples following 24-hour of cisplatin ± SRI-011381 treatment.** (b)** Total cell apoptosis (%) after 24 hours of treatment. **(c)** Percentage of cell proliferation after 24 hours of treatment. # and* represented statistical significances compared to cisplatin and control, respectively. ^#^ p < 0.05, and ^###^ p< 0.001, ** p< 0.01, and **** p< 0.0001.

This study further intended to examine whether TGF-β activation using SRI-011381 would produce a contrary effect compared to the effect of TGF-β blockade on cisplatin cytotoxicity. As observed in previous results, cisplatin treatment significantly increased total apoptosis compared to untreated cells (Fig. 4a and 4b). In addition, the combination treatment of cisplatin and SRI-011381 mitigated cisplatin-induced cell apoptosis. Breast cancer cells treated with SRI-011381 alone showed no change in apoptosis. Furthermore, the cell proliferation of breast cancer cells following treatment with cisplatin with or without SRI-011381 was assessed. The cell proliferation was significantly reduced by cisplatin monotherapy, but this effect was diminished upon combining cisplatin and SRI-011381 (Figure 4c).

### The amount of DNA damage induced by cisplatin was reduced by TGF-β activation using SRI-011381 in breast cancer cells

**Figure 5:**
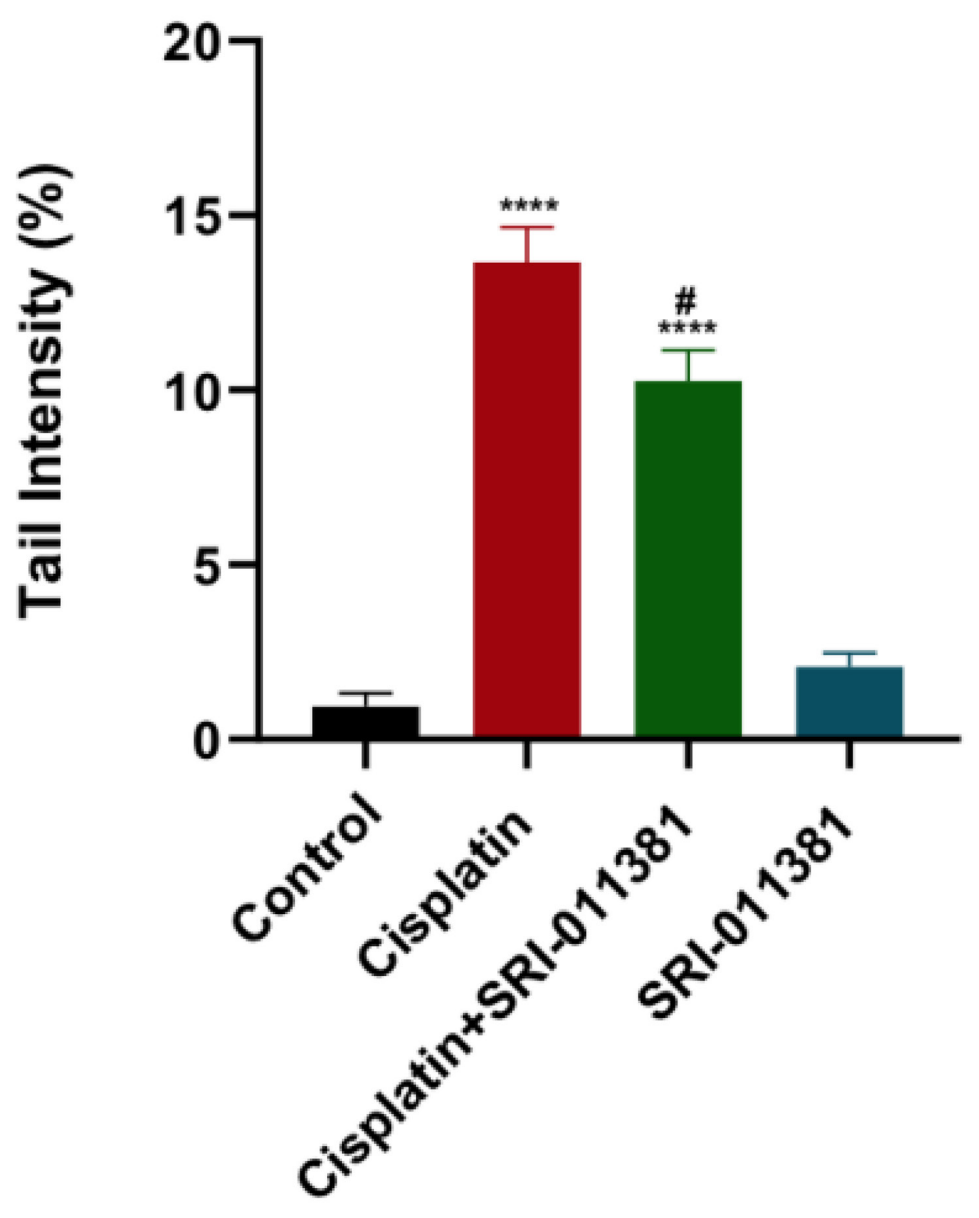
** Influence of SRI-011381 on cisplatin-induced DNA damage.** Amount of DNA damage represented by the tail intensity (%) after treatment with cisplatin ± SRI-011381 for 24-hour. # and* represented statistical significances against cisplatin and control, respectively. ^#^ p < 0.05 and **** p< 0.0001.

As mentioned, cisplatin caused a marked elevation in DNA damage as quantified by COMET assay. Contrary to TGF-β inhibition, DNA damage was significantly reduced upon treating breast cancer cells with SRI-011381 and cisplatin compared to cisplatin alone (Fig. 5). The DNA damage was not affected by SRI-011381 alone.

### TGF-β activation using SRI-011381 upregulated the expression of NER genes induced by cisplatin

**Figure 6:**
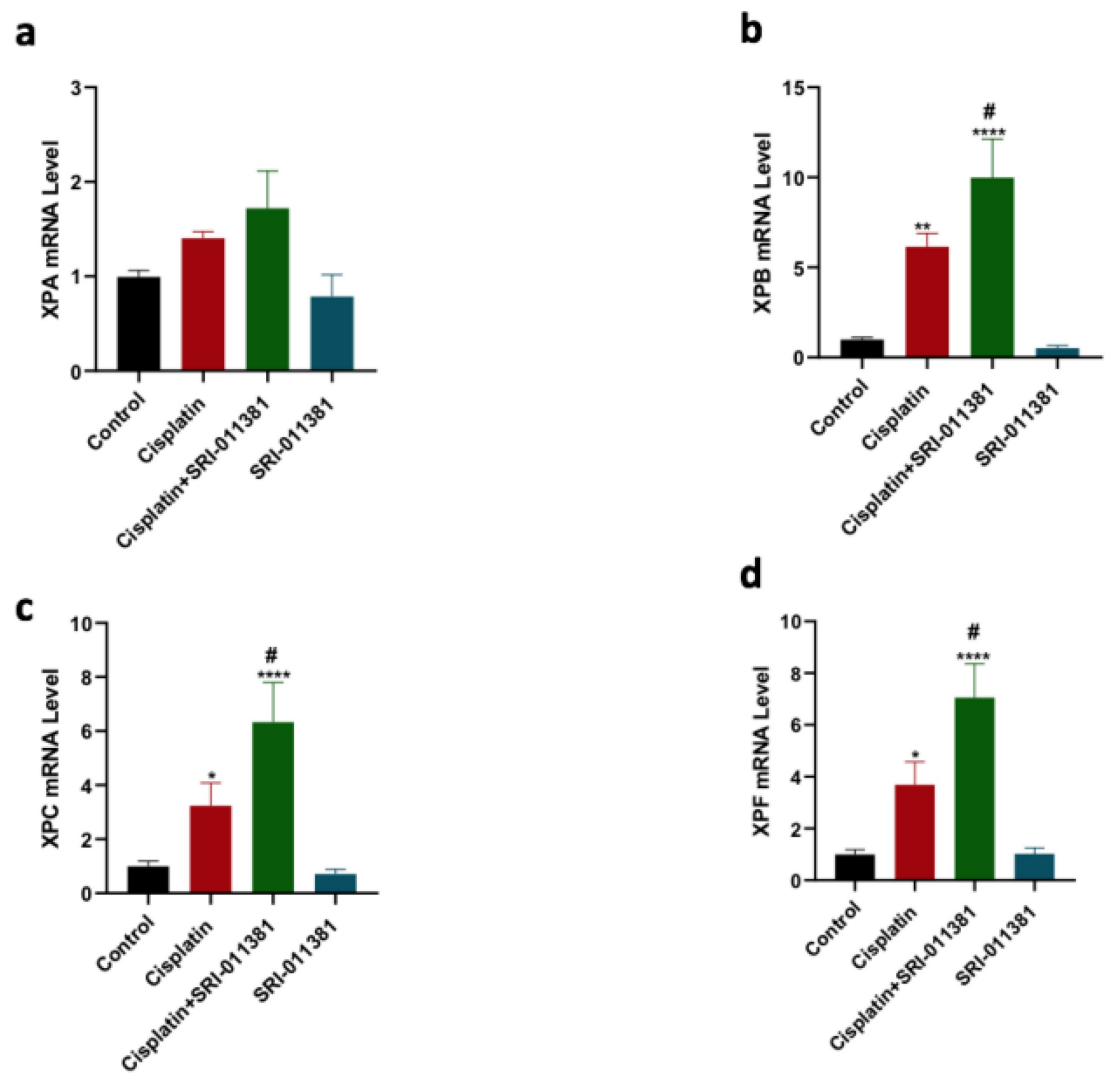
** Influence of SRI-011381 on the Expression of NER Genes Induced by Cisplatin Therapy.** Expression of XPA, XPB, XPC, and XPF genes (Figure 6 a-d) in cells treated with cisplatin ± SRI-011381 for 24 hours. # and* represented statistical significances compared to cisplatin and control, respectively. ^#^p< 0.05, *p< 0.05, ** p< 0.01, and **** p< 0.0001.

The gene expression of XPA, XPB, XPC, and XPF was further evaluated in the context of TGF-β activation and cisplatin therapy using qPCR. Cisplatin therapy significantly increased the expression of XPB, XPC, and XPF genes relative to control (Figure 6 b-d). Cotreatment of cisplatin and SRI-011381 further upregulated the expression of XPB, XPC, and XPF genes compared to cisplatin monotherapy. SRI-011381 alone did not change the expression of XPA, XPB, XPC, and XPF in the breast cancer cells (Figure 6 a-d).

## Discussion

Chemotherapy is a fundamental therapeutic strategy for many types and stages of cancer. Many patients show an initial response to chemotherapy, which changes afterward because of treatment resistance, leading to treatment failure, recurrence, and metastatic progression. Therapy resistance has been reported for all types of chemotherapeutic agents, especially in advanced-stage cancer, where treatment options are limited. Furthermore, chemotherapeutic resistance has been attributed to alterations in various signaling pathways, such DNA repair, tumor microenvironment, drug metabolism, and intracellular signaling pathways 9,23-25. Based on these, the current study designed to identify potential implications of the TGF-β pathway in modulating NER function, thereby inducing breast cancer resistance to cisplatin therapy.

Alterations in the NER pathway can have major consequences on the response to chemotherapy. For example, NER deficiency has been correlated with high sensitivity for cisplatin-based chemotherapy in testicular cancer 26. Polymorphisms in NER genes have also been linked with susceptibility to cisplatin in head, neck, and lung cancer 27,28. Conversely, an association between cisplatin resistance and upregulation of NER genes such as ERCC1, XPF, XPA, and XPD was discovered 29-32. Therefore, elucidating the underlying mechanism of NER-induced chemotherapeutic resistance is needed to advance cancer research, therapy, and outcomes.

Results of this study revealed that cisplatin cytotoxicity was altered by TGF-β signaling modulation as evidenced by increasing and decreasing cytotoxicity with pharmacological TGF-β inhibitor and activator, respectively. LY2109761 was found to enhance the efficacy of cisplatin in xenograft model of ovarian cancer and in-vitro model of parental and cisplatin-resistant ovarian cancer cells 33. Genetic knockdown of TGF-β1 was found to sensitize A549 cancer cells to cisplatin therapy 34. In osteosarcoma, cisplatin efficacy was reduced upon treatment with exogenous TGF-β 35 Additionally, another study had revealed that cisplatin therapeutic effect on non-small lung cancer cells was reduced by treatment with exogenous TGF-β1 36. However, it is still not known whether the cisplatin efficacy could be affected by the TGF- β pathway in breast cancer, which is the focus of our study. Our findings, in line with previous studies, further supported the assertion about the potential role of TGF-β pathway inhibition in improving the chemotherapeutic response of breast cancer cells.

Evidence exists about the interaction between TGF-β pathway and DNA repair processes. TGF-β was rapidly activated by ionizing radiation that induced DNA double-strand damage 16. A knockout mice model of TGF-β1 showed high sensitivity to ionizing radiation as proved by accumulated DNA damage and increased cell death 17. Several studies have also demonstrated that inhibition of TGF-β before ionizing radiation treatment improved cell death and delayed tumor growth 14,18,37. These results suggest that the cellular response to ionizing radiation can be altered by TGF-β pathway via modulating DNA double-strand break pathways.

A new role for TGF-β singling in regulating DNA repair processes has been identified. This study demonstrated a significant impact of TGF-β signaling on DNA damage response, as shown by altering DNA damage response upon combining cisplatin with either pharmacological inhibitor or activator of TGF-β. In this study, the expression of NER genes was assessed 24 hours after cisplatin treatment, as NER activation has been shown to occur within hours in response to cisplatin-induced DNA damage 38,39. Zheng *et* al. revealed that activation of TGF-β signaling enhanced the repair of bulky DNA adducts triggered by the environmental carcinogen, benzo-[a]-pyrene, and photoproducts caused by ultraviolet radiation. This effect was attributed to increased interaction between NER components XPF and XPA with ERCC1 19. Furthermore, a previous study showed that TGF-β1 genetic knockdown potentiated cisplatin efficacy on human A549 non-small lung cancer cells via reducing drug-resistant proteins, including NER protein, ERCC1 34. Upregulation of miR187 in gastric cancer significantly downregulated the protein expression of Smad4, TGF-β1, and NER components (ERCC3 and ERCC4), which boosted the cisplatin sensitivity. Additionally, the protein expression of Smad4, TGF-β1, ERCC3, and ERCC4 were increased by miR187 inhibition, suggesting a feasible link between TGF-β and NER pathways 40. Furthermore, loss of Smad4, a TGF-β signaling component, in keratinocytes impaired the repair of DNA damage induced by ultraviolet that is mainly repaired by NER. Interestingly, the ERCC1 gene was also significantly downregulated in keratinocytes with Smad4 deletion compared to wild-type keratinocytes 41. Oppositely, inhibition of E-cadherin impaired the repair of ultraviolet radiation-induced DNA damage along with suppression of XPC and DNA damage-binding protein 1 (DDB1) level via activation of TGF-β signaling 42. Collectively, these data indicate a potential implication of TGF-β pathway in modulating NER and, thereby, chemotherapeutic response.

While our study demonstrated the connection between TGF-β pathway, NER, and cisplatin efficacy in breast cancer, it remains unclear whether this effect can also be observed in other types of cancer. Furthermore, there is a need to conduct similar study with other anticancer agents to determine if the effect is exclusive to cisplatin or can be generalized to different chemotherapeutic agents. The effect of genetic knockdown of TGF-β pathway-associated genes on the NER pathway and cisplatin sensitivity could also be evaluated in upcoming studies.

To conclude, this study provided evidence for the TGF-β pathway contribution to altering the response to cisplatin therapy. Furthermore, our data will further help to understand the mechanisms of chemotherapeutic resistance in cancer that can eventually help to create a novel approach to overcome resistance and improve treatment outcomes.

## Figures and Tables

**Table 1:** Human forward and reverse primer sequences

Gene Symbol	Forward Primer	Reverse Primer
XPA	5′-CCGACAGGAAAACCGAGAAA-3′	5′-TTCCACACGCTGCTTCTTACTG-3′
XPB	5′-CAAAAGCATGGTGCTGAGTG-3′	5′-CCACTTCTGGCAACCACTGA-3′
XPC	5′-CCCAGCCCGCTTTACCA-3′	5′-TGCATTAACTGTAAATGTTCCAATGA-3′
XPF	5′-CACCTCCCTCGCCGTGTA-3′	5′-CGCAAATATAACACCACCTTGTG-3′
GAPDH	5′-GCCAAGGTCATCCATGACAACT-3′	5′-GAGGGGCCATCCACAGTCTT-3′
